# Assessment of the distribution of load on the lower limbs and balance before and after ankle arthrodesis with the Ilizarov method

**DOI:** 10.1038/s41598-018-34016-3

**Published:** 2018-10-24

**Authors:** Piotr Morasiewicz, Grzegorz Konieczny, Maciej Dejnek, Wiktor Urbański, Szymon Łukasz Dragan, Mirosław Kulej, Szymon Feliks Dragan, Łukasz Pawik

**Affiliations:** 1 Wroclaw Medical University, Department and Clinic of Orthopaedic and Traumatologic Surgery, Borowska 213, 50-556 Wrocław, Poland; 2 Faculty of Health Sciences and Physical Education, Witelon State University of Applied Sciences in Legnica, Sejmowa 5A, 59-220 Legnica, Poland; 3 University of Physical Education, Department of Physiotherapy and Occupational, Therapy in Motor Disorders and Dysfunctions, Al. IJ Paderewskiego 35, 51-612 Wrocław, Poland

## Abstract

Ankle arthrodesis with the Ilizarov method is an accepted form of treatment of advanced degenerative changes of the ankle joint. Incorrect balance and load distribution on the lower limbs may result in pain and dysfunction. The aim of the study was to assess the change of balance and load distribution in lower extremities in patients before and after ankle arthrodesis with the Ilizarov method. Between 2013 and 2016, ankle arthrodesis using the Ilizarov method was performed on 21 patients. The evaluation of balance and percentage of load in each lower limb was performed before the surgery and during the follow-ups. The evaluation was performed using a Zebris pedobarographic platform. Before the surgery, the patients exhibited an average load of 41.9% of body weight in the affected limb, whereas the load in the healthy limb was 58.1%. The difference was statistically significant (p = 0,000031). In two years follow-up, the average load in the treated limbs was 48.19%, whereas the healthy limbs were subjected to an average load of 51.81%. In preoperative tests, the average path length of the center of gravity was 161.55 cm; postoperatively, the average path length of the center of gravity was 129.7 cm (p = 0.00003206). Preoperatively, the average area of the center of gravity was 18.85 cm^2^; it decreased to 6.19 cm^2^ (p = 0.000032) postoperatively. Arthrodesis of the ankle with the Ilizarov method improved the statics of the musculoskeletal system by improving the distribution of loads in the lower limbs as well as balance. However, it failed to restore the parameters of a healthy person. Advanced degenerative changes of the ankle disturb the biomechanics of the entire lower limb.

## Introduction

Degenerative changes of the ankle joint are a substantial problem. It is estimated that about 50,000 patients are diagnosed with ankle osteoarthrosis (OA) each year^1^, mainly of post-traumatic etiology^2,3^. Advanced ankle OA and deformations result in pain, restrained mobility, muscle weakness and impaired sport performance^1,4–9^. Ankle arthrodesis with the Ilizarov method is an accepted form of treatment of severe ankle OA, especially when the former is associated with deformity, limb shortening, poor bone and soft tissue quality^1,8–15^. Ankle arthrodesis reduces or eliminates the pain, aligns the ankle in the correct position and improves limb function; however, it results in restricted ankle movement^1,8–15^. Patients after arthrodesis report reduced pain and exhibit improved quality of life scores; however, they also report reduced mobility and difficulties with everyday activities^1,9–15^. The aim of the surgery is to reduce the pain and improve the limb function^1,11–15^. Incorrect balance and load distribution between lower limbs may result in pain and dysfunction^1,6–8,16–18^, which may worsen the results of treatment and reduce the quality of life^1,18^. Therefore, it is important to assess the lower limbs from the biomechanical point of view in patients before and after ankle arthrodesis.

The biomechanics of the lower limb in patients after ankle arthrodesis has not been fully understood. Many authors have focused on the assessment of gait after ankle arthrodesis^9,19,20^. However, there are no papers prospectively evaluating the statics of the musculoskeletal system after ankle arthrodesis using the Ilizarov method. Wu and Barito observed a lack of full normalization of gait after ankle arthrodesis with internal fixation^9,19^. On the other hand, Tenenbaum reported an improvement of gait parameters after ankle arthrodesis^20^. Pedobarographic analysis allows for an objective analysis of the static and dynamic conditions of the lower limb before and after the surgery^6–8,16,17,21,22^.

The aim of this study was to assess the balance and distribution of loading forces between the lower extremities in patients before and after ankle arthrodesis with the Ilizarov method.

## Material and Methods

Between 2013 and 2016, ankle arthrodesis with the Ilizarov method was performed in 21 patients at our department. Advanced osteoarthrosis and deformity of the ankle were the indications for the procedure. The criteria for inclusion in the study were as follows: a history of ankle arthrodesis with the Ilizarov method, availability of results of pedobarographic analysis before and after the surgery, availability of radiological analysis and epidemiological data before and after the surgery, at least 2 years of follow-up after the surgery/treatment, no other comorbidities of the lower limbs, no previous surgeries of the lower limbs, consent to participate in the study. The criteria for exclusion from the study were: lack of ankle arthrodesis using the Ilizarov method, lack of results of pedobarographic studies before and after the surgery, lack of radiological analysis and epidemiological data before and after the surgery, a period of observation shorter than 2 years after the conclusion of treatment, lack of consent to participate in the study, accompanying diseases of the lower limbs, other surgeries performed on the lower limbs. There were no cases of bilateral arthrodesis in our material. The study had been approved by the local bioethics committee. All participants had been informed about the study and participated voluntarily. Informed consent was obtained from the participants and the parents/legal guardians of under-18 patient(s) that participated in the study.

The Ilizarov external fixator used in the procedure consisted of two rings attached to the lower leg with Kirschner wires, and one U-shaped foot ring attached to the calcaneus and metatarsus using Kirschner wires equipped with supplementary olives. Ankle arthrodesis was performed with anterior approach. The compression necessary for ankle arthrodesis was obtained on the operating table. The patients started to bear weight on the first postoperative day^10,23^, with full load as soon as they tolerated it. The Ilizarov apparatus remained attached for at least 9 weeks post-surgery. Radiological bone union, defined as complete cortical or callus bridging, trabeculation across the ankle joint and loss of lucency between fusion surfaces, and clinical union, defined as no pain and motion when stress is applied to the ankle joint during clinical examination, were an indication for fixator removal^10^. After removing the Ilizarov fixator, the patients were supplied with a stiff ankle-foot orthosis for 6 weeks. In approximately 3 months post-surgery, the patients were allowed to walk in regular footwear^10,23^. All the procedures were performed exclusively by two orthopedic surgeons.

The evaluation of balance and percentage of load distribution between the lower limbs was performed before the surgery and in post-surgery follow up after at least 2 years. The evaluation was performed using a pedobarographic platform (Zebris Medical, Gmbh)^6–8^. The platform, sized 320 × 470 mm and equipped with 1504 sensors, was connected to a PC. Foot Print software (version 1.2.4.9) was used to record and process the data from the pedobarographic platform. The tests of balance and load distribution were performed with eyes open and barefoot. Before each test, the device was calibrated and the patient was informed about the test method. The test was carried out for 90 seconds in a bipedal position. Each patient underwent the test three times and the average scores of these measurements were recorded for further analysis^6–8^.

**Figure 1:**
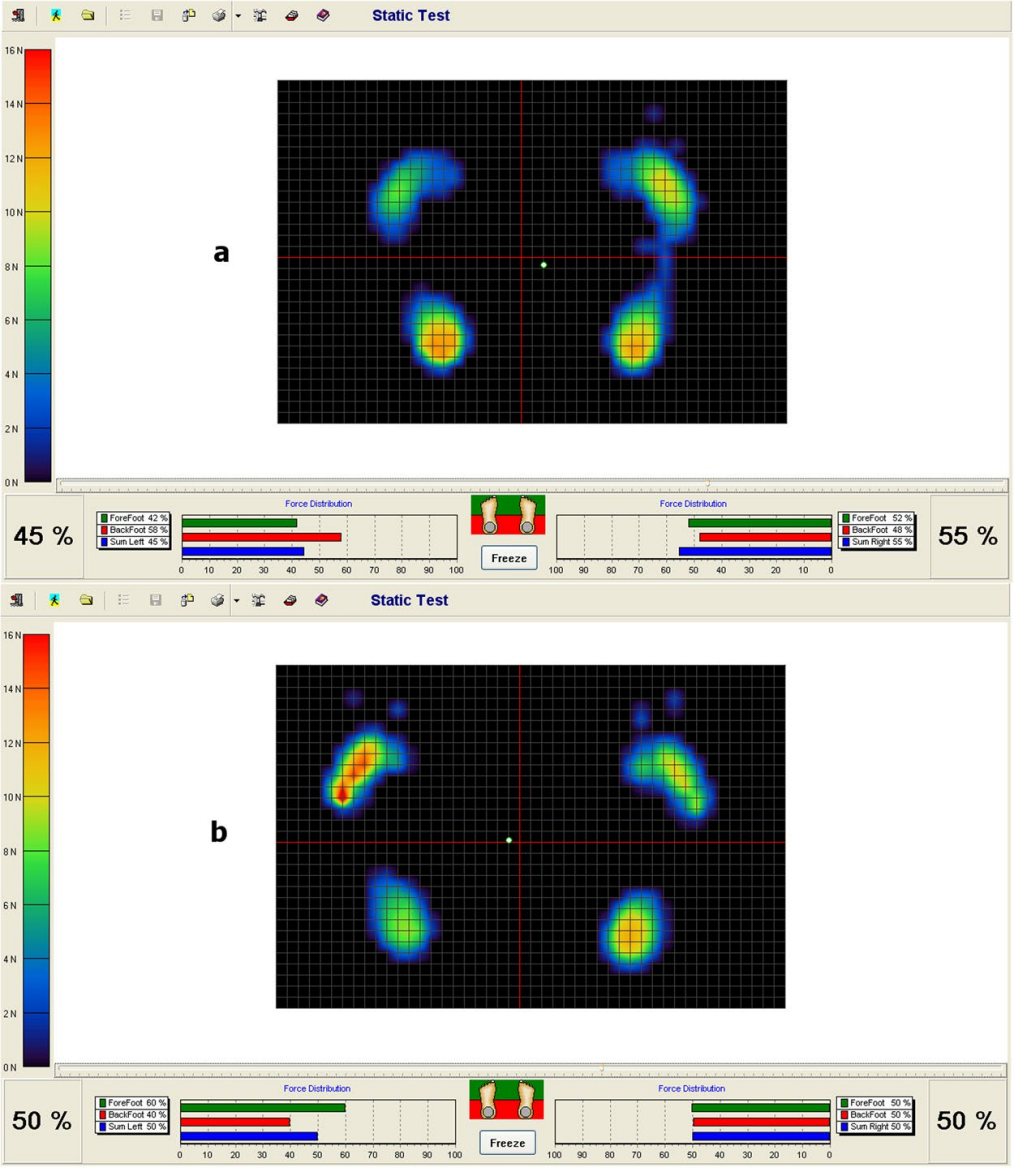
(**a**,**b**) Distribution of load on the operated and healthy limb (**a**: preoperative status, **b**: postoperative improvement).

**Figure 2:**
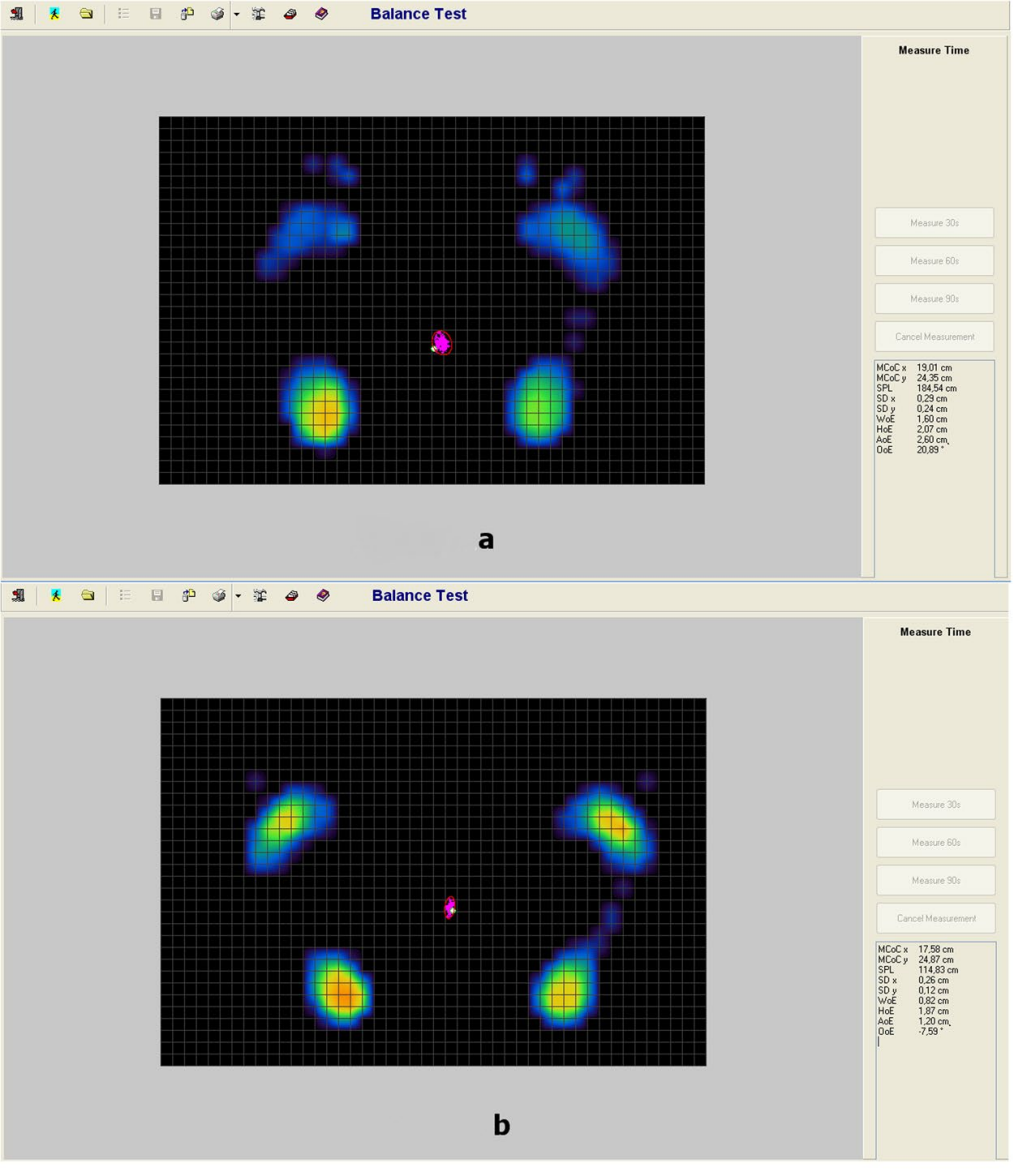
(**a**,**b**) Path length of the center of gravity and area of the center of gravity (**a**: preoperative status, **b**: postoperative improvement).

Load distribution between the operated and healthy limb was expressed as a percentage (Fig. 1a,b). Balance was described as a distance from the center of gravity (the length of the center of gravity line created during the measurement) indicated in centimeters (cm). It was also described as an area of the center of gravity (the surface area of the position of the center of gravity created during the measurement), expressed in square centimeters (cm^2^) (Fig. 2a,b)^6–8^.

All statistical analyzes were prepared with the statistical package R version 3.1.1. A level of significance of 5% was adopted for testing the statistical hypotheses. Each time, one-sided Wilcoxon tests were used to test the statistical hypotheses.

### Ethical approval and informed consent

All experiments were carried out in accordance with relevant guidelines and regulations. The study was approved by the Bioethics Commission of Wroclaw Medical University.

## Results

The treated group consisted of 21 patients (13 males and 8 females) who had undergone ankle arthrodesis with the Ilizarov method. Bone union was achieved in all 21 treated patients. The average age was 38 years and 8 months (16 years and 7 months–67 years and 10 months). The etiology of ankle arthritis was traumatic in 15 cases, inflammatory in 5 cases and innate in 1 case. The observation period was 33 months on average (25–60).

**Figure 3:**
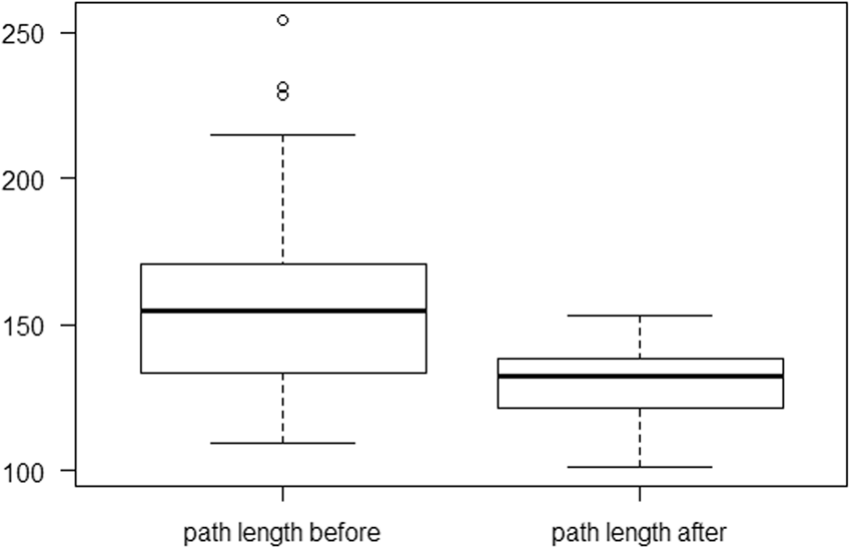
Average path length of the center of gravity before and after the surgery.

**Figure 4:**
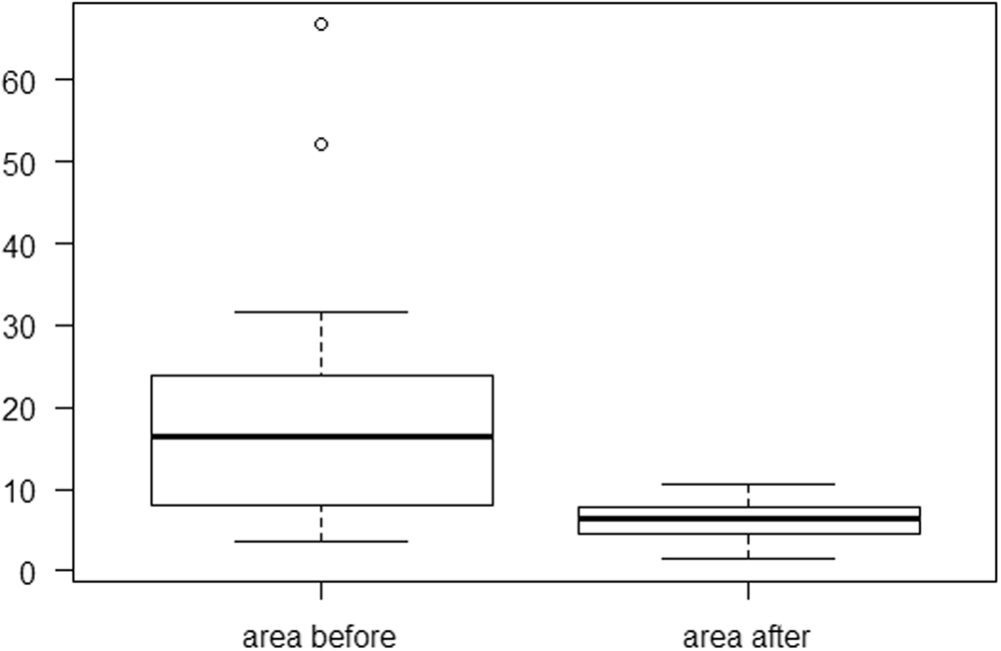
Average area of the center of gravity before and after the surgery.

Before the surgery, the group of respondents exhibited an average load of 41.9% (SD of 6.49) of the body weight on the affected limb, while the load on the healthy limb was 58.1% (SD of 6.49). The difference was statistically significant (p = 0.000031). In two years follow-up, the average load on the affected limb changed and reached 48.19% (SD of 3.17) of the body weight, while in the healthy limb the average load decreased to 51.81% (SD of 3.17). The difference, however, was not statistically significant (p = 0.06765). The difference between the average load on the affected limb before and after the surgery was statistically different (p = 0.000047). While comparing the load on the healthy limb, statistically significant differences (p = 0,000047) were identified in tests before and after the surgery. Preoperatively, the average distance from the center of gravity was 161.55 cm (SD – 40.09), while in two years follow-up it decreased to 129.7 cm (SD 14.28) (Fig. 3), and the results were statistically significant (p = 0.00003206). In the analyzed group of patients, the average area of the center of gravity decreased significantly, from 18.85 cm^2^ (SD – 16.32) preoperatively to 6.19 cm^2^ (SD – 2.51) postoperatively (Fig. 4). The differences were statistically significant (p = 0,000032).

## Discussion

Injuries are one of the most common causes of advanced osteoarthritis and deformities of the ankle^1–3,10,23^. Limited ankle function, mobility and sport performance are caused by pain, restricted motion, and deformity or instability^1,4,5,9–15,23^. Arthrodesis is a common method of treatment of advanced ankle destruction and deformity^1,10–15,23^. Fixing the joint is beneficial because it removes pain, instability and deformity. On the other hand, it immobilizes the joint in a position which is not always optimal in relation to the other joints.

The biomechanics of the lower limb after ankle arthrodesis has not yet been fully studied. Wu *et al*. identified a restriction of the foot’s range of motion (ROM) in walking patients after ankle arthrodesis with internal fixation^9^. Braito noted an asymmetry of gait and restricted mobility in patients after ankle arthrodesis and ankle endoprosthesis^19^.

No reports are available in the literature assessing the statics of the musculoskeletal system – the balance and load distribution in the lower limbs and how they change after ankle arthrodesis using the Ilizarov method. The authors of the paper prepared an article for publication, compared the statics of musculoskeletal systems after ankle arthrodeses using the Ilizarov method and internal fixation, but they assessed the postoperative results only. In previous papers, the authors assessed the balance and load distribution on the lower limbs before and after the surgery in patients who had undergone corticotomies using the Ilizarov method^8^, after cortices using the Ilizarov method in comparison with a group of healthy volunteers^7^, depending on the type of osteotomy using the Ilizarov method^6^, and in patients at the ward – several days after osteotomy using the Ilizarov method^22^.

In the present study, we have noted a significant improvement of balance and distribution of loads in the lower limbs in patients after ankle arthrodesis using the Ilizarov method. Deformity, pain and restricted ROM affect the distribution of loads in the lower limbs^16,17,21^. Symmetrical distribution of loads between the lower limbs is important for proper biomechanics^16,17,21^. Rongies *et al*. found a correlation between reduced pain and improved balance and evener distribution of loads in the lower limbs in patients with coxarthrosis^17^, which could be due to improved proprioception. In patients included in this study, the authors achieved a nearly symmetrical distribution of loads in lower limbs after ankle arthrodesis with the Ilizarov method. The results were similar to the results obtained from a control group of healthy volunteers presented in the author’s previous study^7^. In the analyzed group, balance after ankle arthrodesis also improved; the parameters were similar to those obtained after osteotomy with the Ilizarov method^8^; however, they were worse than the results achieved by the group of healthy volunteers^7^.

Queen *et al*. demonstrated that patients with ankle OA suffer from significant locomotor disorders^1^. The movement of their toes is restricted, the knee and hip ROM is impaired and the biomechanics of the neighboring joints is disturbed^1^. The above-mentioned symptoms disturb the balance and may increase the risk of falls in patients with ankle OA^1,18^. Ankle arthrodesis with the Ilizarov method has a positive impact on the biomechanics of the musculoskeletal system; however, surgical intervention concerns only the ankle and does not involve the knee or the hip. This may be the reason behind the improvement of balance parameters which, however, do not match those of the healthy volunteers.

Long-term disorders of the foot and ankle may cause the central nervous system to adapt to the pathological conditions^21^ and lead to the formation of compensatory locomotor mechanisms^5,7–9^. This may limit the possibility of restoring the correct parameters of balance after ankle arthrodesis. It is not only the statics but also the dynamics of the musculoskeletal system that may not be fully normalized after ankle arthrodesis. Wu and Barito have observed a lack of full normalization of gait parameters after ankle arthrodesis with internal fixation^9,19^.

Patients after ankle arthrodesis report increased walking speed and reduced pain. Mobility, however, does not improve^1^ and the authors believe that failure to achieve the parameters of a healthy person may be the reason. Other methods of treatment of advanced ankle OA also fail to provide a full recovery of balance^1^. In a group of patients after ankle endoprosthesis, Queen observed even worse balance parameters after the surgery than before it^1^.

Evaluation using a pedobarographic platform is fast, simple and non-invasive^6–8,16,17,21^; it makes it possible to assess the outcome of the treatment and to determine the potential need for further treatment.

Limitations of this paper include the small group of patients and the length of the follow-up. Due to the limited time frame, we did not gather a larger group of patients after ankle arthrodesis using the Ilizarov method and we do not possess a longer observation period. However, most authors also have a similar number of patients^12,15,19,20,23^ with a similar observation period^15,19,20,23^. According to the authors of this study, pedobarographic gait evaluation in patients after ankle arthrodesis with Ilizarov method gives significant information regarding the patients’ outcome and the authors recommend its use in the future.

Arthrodesis of the ankle with the Ilizarov method improves the statics of the musculoskeletal system. Symmetrical distribution of loads in the lower limbs was achieved after the treatment. Balance improves after ankle arthrodesis with the Ilizarov method, but it does not recover fully to the parameters of a healthy person. Advanced osteoarthritis of the ankle alters the biomechanics of the entire lower limb.

## Data Availability

The datasets used and analyzed during the current study are available from the corresponding author on reasonable request.
